# Causal associations between 1400 circulating metabolites and spontaneous abortion: A bidirectional 2-sample Mendelian randomization study

**DOI:** 10.1097/MD.0000000000045981

**Published:** 2025-11-21

**Authors:** Xuan Zhou, Yu-Xuan Fang, Da-Wei Zhang, Li-Ya Ma, Man-Man Yao

**Affiliations:** a Henan University of Chinese Medicine, Zhengzhou, China; b Department of Gynecology, Third Affiliated Hospital of Henan University of Chinese Medicine, Zhengzhou, China; c Department of Gastroenterology, The First Affiliated Hospital of Henan University of Chinese Medicine, Zhengzhou, China.

**Keywords:** causal inference, Mendelian randomization, metabolism, metabolites, spontaneous abortion

## Abstract

Spontaneous abortion (SA) affects approximately 10% to 15% of clinically recognized pregnancies. The potential causal role of metabolic disturbances in SA remains poorly defined. We performed a bidirectional 2-sample Mendelian randomization (MR) analysis to evaluate causal associations between 1400 plasma metabolites and SA. Genetic instruments for metabolites were obtained from a genome-wide association study of 8299 individuals from the Canadian longitudinal study on aging. Summary statistics for SA were derived from the FinnGen R12 dataset, including 23,167 cases and 1,99,279 controls. Primary MR analyses used the inverse variance weighted method, with MR-Egger, weighted median, Mendelian Randomization Pleiotropy RESidual Sum and Outlier, and leave-one-out analyses conducted to assess robustness. Thirty-six circulating metabolites showed significant causal associations with SA. Key findings included protective associations for phenylacetylglutamine (odds ratio [OR], 0.89; 95% confidence interval [CI], 0.83–0.96; *P* = .0019) and risk-enhancing effects for the glycerol-to-carnitine ratio (OR, 1.07; 95% CI: 1.03–1.12; *P* = .0021), 1-methyl-5-imidazoleacetate (OR, 1.09; 95% CI: 1.03–1.16; *P* = .0040), and the caffeine-to-theophylline ratio (OR, 1.08; 95% CI: 1.03–1.15; *P* = .0041). Sensitivity analyses showed no evidence of horizontal pleiotropy or heterogeneity. Reverse MR identified 5 metabolites potentially influenced by genetic predisposition to SA, though forward causal effects were more prominent. This study provides genetic evidence supporting a causal role of specific circulating metabolites in the pathophysiology of SA. These findings offer novel mechanistic insights into early pregnancy loss and highlight potential biomarkers for reproductive risk stratification and targeted intervention.

## 1. Introduction

Spontaneous abortion (SA), defined as the unintended loss of pregnancy before 20 weeks of gestation, affects approximately 10% to 15% of clinically confirmed pregnancies and remains a major contributor to reproductive failure among women of reproductive age.^[1]^ While chromosomal abnormalities, endocrine disorders, and anatomical anomalies account for some cases, a substantial proportion of SA events are idiopathic, underscoring the need to identify novel biological contributors.^[2]^

Recent studies suggest that systemic metabolic homeostasis plays a critical role in early pregnancy maintenance.^[3,4]^ Circulating metabolites – small molecules that reflect the integrated effects of genetic, environmental, and physiological factors – have shown promise as biomarkers for reproductive outcomes. However, most existing studies investigating metabolite – SA associations are observational in nature and thus susceptible to confounding and reverse causality, limiting their capacity to infer causation.^[5]^

Mendelian randomization (MR) provides a robust framework to address these limitations by leveraging genetic variants as instrumental variables (IVs) to estimate causal effects between exposures and outcomes.^[6]^ Two-sample MR extends this approach by utilizing genome-wide association study (GWAS) summary statistics from independent cohorts, thereby improving statistical power and generalizability.^[7]^ Furthermore, bidirectional MR enables the evaluation of reverse causality, which is particularly relevant in biological processes involving feedback mechanisms, such as metabolism during pregnancy.^[8]^

In this study, we performed a comprehensive bidirectional 2-sample MR analysis to investigate the causal relationship between circulating plasma metabolites and SA. Genetic instruments for 1091 plasma metabolites and 309 metabolic ratios were derived from a large-scale GWAS involving 8299 individuals of European ancestry from the Canadian longitudinal study on aging, conducted by Chen et al^[9]^ Summary-level data for SA were obtained from the Finn Gen R12 release (https://www.finngen.fi/), comprising 23,167 cases and 1,99,279 controls. By integrating these datasets, we aimed to: identify circulating metabolites with causal effects on SA risk; assess potential reverse effects of SA on systemic metabolism; and provide insights into metabolic mechanisms underlying pregnancy loss.

## 2. Materials and methods

### 2.1. Study design

**Figure 1.**
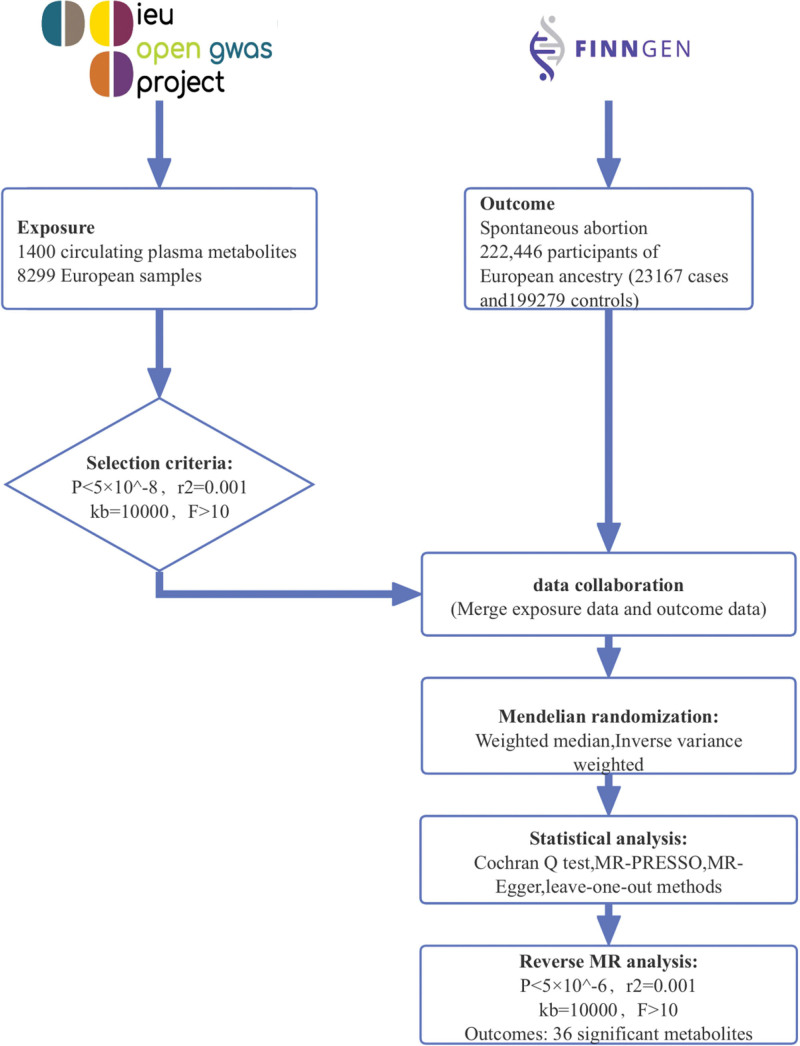
Study flowchart depicting the selection process of plasma metabolites and genetic instruments for MR analysis. This figure outlines the data curation, SNP selection, and analytical steps involved in deriving the causal inferences between plasma metabolites and SA. MR = Mendelian randomization, SA = spontaneous abortion, SNP = single-nucleotide polymorphism.

A bidirectional 2-sample MR analysis was performed to investigate the causal relationship between 1400 circulating plasma metabolites and SA. In the forward MR direction, plasma metabolites were treated as exposures and SA as the outcome. For the reverse analysis, only those metabolites that showed significant associations in the forward MR were included as outcomes, with SA considered as the exposure. To ensure the robustness of the findings, we performed pleiotropy, heterogeneity, and sensitivity analyses. An overview of the study design is shown in Figure 1.

### 2.2. MR analysis and instrumental variable selection

We employed a bidirectional MR framework to evaluate causal relationships between circulating plasma metabolites and SA. Single-nucleotide polymorphisms (SNPs) were selected as IVs based on the following core assumptions:

1. The IVs are robustly associated with the exposure;
2. The IVs affect the outcome exclusively through the exposure and not via alternative pathways;
3. The IVs are independent of confounders.

For the forward MR analysis, SNPs associated with plasma metabolites were selected at a genome-wide significance threshold of *P* < 5 × 10⁻⁸. In the reverse MR analysis, a more lenient threshold of *P* < 5 × 10⁻⁶ was applied, considering the limited number of genome-wide significant variants associated with SA. Linkage disequilibrium (LD) pruning was performed using *r²* < 0.001 and a window size of 10,000 kb. Only SNPs with *F*-statistics > 10 were retained to minimize weak instrument bias.

To further ensure instrument validity, we excluded SNPs known to be associated with potential confounders using the PhenoScanner V2 database (http://www.phenoscanner.medschl.cam.ac.uk/).

Primary causal estimates were obtained using the inverse variance weighted (IVW) method. To assess the robustness of the findings, we performed sensitivity analyses using the weighted median estimator (WME), MR-Egger regression, single SNP analysis, and leave-one-out analysis. All MR methods were applied consistently in both forward and reverse directions. Associations with *P* < .05 in both IVW and WME were considered robust. All analyses were conducted using the TwoSampleMR package in R version 4.3.1.

### 2.3. GWAS data sources for metabolites

Genetic summary statistics for 1091 plasma metabolites and 309 metabolite ratios were obtained from a large-scale GWAS conducted by Chen et al (PubMed ID: [PMC7614162]). The study included 8299 individuals of European ancestry from the Canadian longitudinal study on aging, and analyzed approximately 15 million SNPs. The full summary-level GWAS data are publicly available via the GWAS catalog (accession numbers: GCST90199621 to GCST90204063).

### 2.4. GWAS data for spontaneous abortion

Summary-level GWAS data for SA were obtained from the 12th release of the FinnGen Consortium (https://www.finngen.fi/en), comprising 23,167 cases and 1,99,279 controls, all of European ancestry. SA cases were defined using physician-recorded international classification of diseases (ICD) codes (ICD-8: 643, ICD-9: 634, ICD-10: O03) from the Finnish national healthcare registers in the FinnGen study. All participants provided informed consent. As only de-identified, publicly available data were used in this study, additional ethical approval was not required.

### 2.5. Sensitivity analysis

To assess the robustness of the causal estimates, a series of sensitivity analyses were performed. The MR-Egger intercept test was used to detect the presence of horizontal pleiotropy, with *P* < .05 considered indicative of potential bias. Heterogeneity across SNP instruments was evaluated using Cochran’s *Q* test and the Mendelian Randomization Pleiotropy RESidual Sum and Outlier (MR-PRESSO) global test (*P* > .05 indicating no significant heterogeneity). Leave-one-out analysis was conducted to examine whether any single SNP disproportionately influenced the overall causal estimate. In addition, funnel plots were generated to visually assess asymmetry, which may suggest potential bias or publication effects.

### 2.6. Reverse MR analysis

In the reverse MR analysis, SA was considered as the exposure. SNPs associated with SA were selected using a significance threshold of *P* < 5 × 10⁻⁶, and LD pruning was performed with *r²* < 0.001 and a window size of 10,000 kb. Each of the 36 metabolites identified as significant in the forward MR analysis was treated as an outcome. The same analytical methods – including IVW, WME, MR-Egger regression, weighted mode, and simple mode – were applied to assess potential reverse causal effects.

## 3. Results

### 3.1. Causal effects of circulating metabolites on spontaneous abortion

Based on the IVs and GWAS datasets described in the section 2, we conducted a 2-sample MR analysis to investigate the causal relationships between 1400 circulating plasma metabolites and SA.

Initial screening using the IVW method identified 45 metabolites that were significantly associated with SA (*P* < .05). After applying further filtering criteria – namely, statistical significance in IVW analysis (*P* < .05), directionally consistent odds ratios (ORs) across all MR methods, and no evidence of horizontal pleiotropy based on the MR-Egger intercept (*P* > .05) – a total of 36 metabolites were retained as robust candidates.

**Figure 2.**
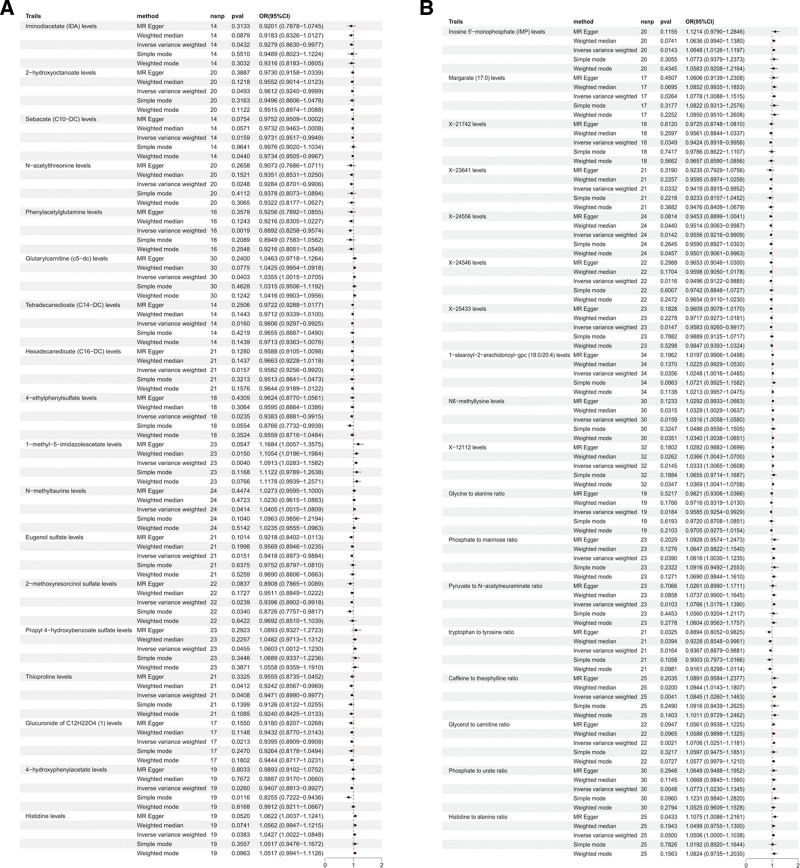
Effect estimates of 36 plasma metabolites across 5 MR methods. Each point represents the causal estimate of a metabolite obtained from MR-Egger, weighted median, IVW, simple mode, and weighted mode methods. Estimates are presented as ORs with corresponding 95% CIs. CI = confidence interval, IVW = inverse variance weighted, MR = Mendelian randomization, OR = odds ratio.

**Figure 3.**
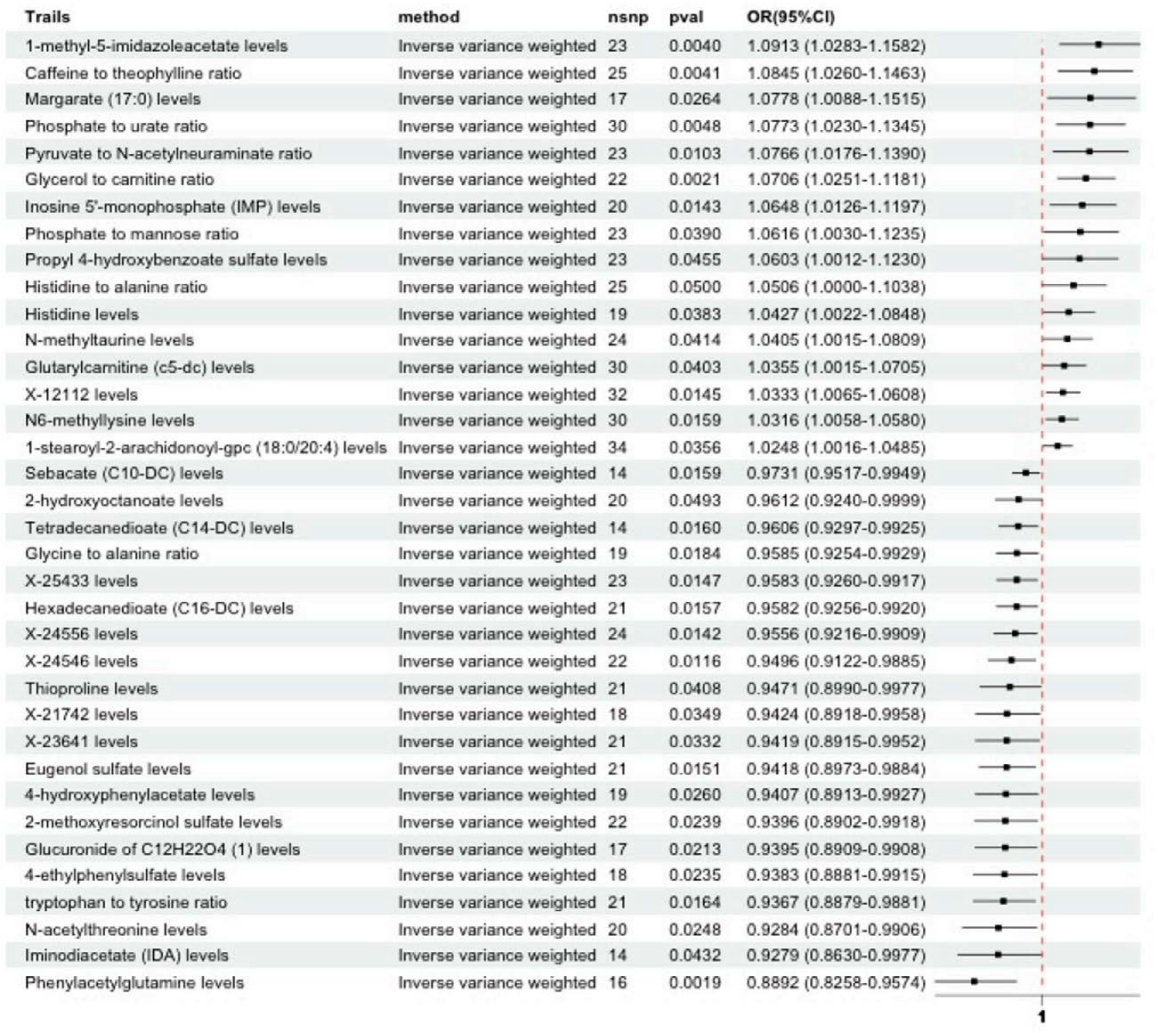
Forest plot of IVW causal estimates for the 36 plasma metabolites associated with SA. Each row indicates a metabolite with corresponding OR and 95% CI. Metabolites are ranked by statistical significance. CI = confidence interval, IVW = inverse variance weighted, OR = odds ratio, SA = spontaneous abortion.

Figure 2 presents the effect estimates of these 36 metabolites using 5 MR methods, including IVW, MR-Egger, weighted median, simple mode, and weighted mode. A forest plot summarizing the IVW-derived ORs and 95% confidence intervals (CIs) for all 36 metabolites is shown in Figure 3.

Among the 36 metabolites, phenylacetylglutamine (PAGln; OR = 0.89, 95% CI: 0.83–0.96, *P* = .0019), glycerol to carnitine ratio (OR = 1.07, 95% CI: 1.03–1.12, *P* = .0021), 1-methyl-5-imidazoleacetate (OR = 1.09, 95% CI: 1.03–1.16, *P* = .0040), and caffeine to theophylline ratio (OR = 1.08, 95% CI: 1.03–1.15, *P* = .0041) exhibited the most significant associations with SA.

The complete list of significant metabolites and their corresponding MR estimates is provided in Table S1, Supplemental Digital Content, https://links.lww.com/MD/Q672.

### 3.2. Sensitivity and visualization analyses

To evaluate the robustness of the MR findings, we conducted multiple sensitivity analyses. The weighted median method yielded results largely consistent with IVW. Cochran’s *Q* test indicated no significant heterogeneity across SNP instruments under both the IVW and MR-Egger models (*P* > .05), as reported in Table S2, Supplemental Digital Content, https://links.lww.com/MD/Q672. No evidence of horizontal pleiotropy was detected based on the MR-Egger intercept test results (*P* > .05 for all), as shown in Table S3, Supplemental Digital Content, https://links.lww.com/MD/Q672. Furthermore, the MR-PRESSO global test did not detect any outlier SNPs among the 36 metabolites (*P* > .05 for all), as summarized in Table S4, Supplemental Digital Content, https://links.lww.com/MD/Q672.

**Figure 4.**
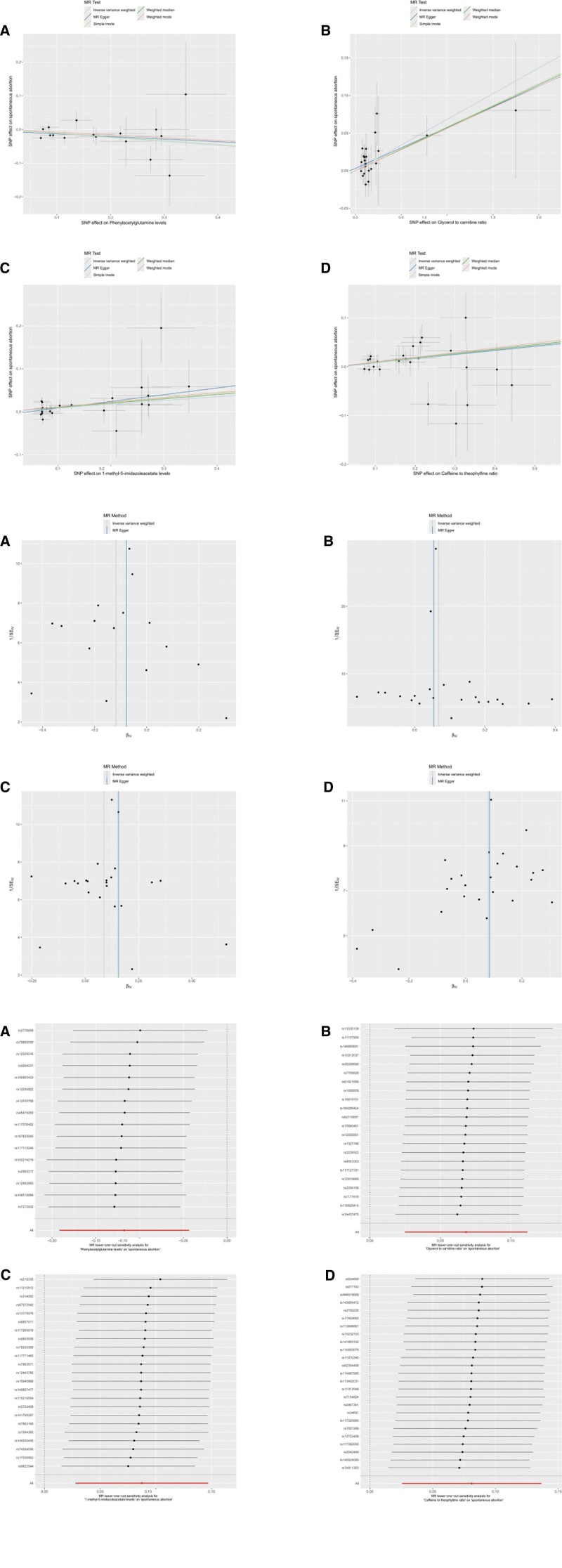
(A**–**C) Visualization of MR analyses for 4 representative metabolites. For each metabolite, 3 diagnostic plots are presented: (A) Funnel plot to assess horizontal pleiotropy, (B) scatter plot of SNP-exposure versus SNP-outcome associations, and (C) leave-one-out sensitivity analysis plot to evaluate the influence of individual SNPs. MR = Mendelian randomization, SNP = single-nucleotide polymorphism.

To evaluate the robustness and directionality of the MR findings, we generated 3 types of diagnostic plots for 4 representative metabolites: PAGln, glycerol to carnitine ratio, 1-methyl-5-imidazoleacetate, and caffeine to theophylline ratio. These included scatter plots (Fig. 4A), funnel plots (Fig. 4B), and leave-one-out sensitivity plots (Fig. 4C).

As shown in Figure 4A, PAGln exhibited a negative causal estimate (panel A), suggesting a potential protective effect against SA. In contrast, the other 3 metabolites – glycerol to carnitine ratio (panel B), 1-methyl-5-imidazoleacetate (panel C), and caffeine to theophylline ratio (panel D) – showed positive slopes, indicating an increased risk of miscarriage. These associations were consistent across MR methods, including IVW, MR-Egger, weighted median, weighted mode, and simple mode. Funnel plots (Fig. 4B) were generally symmetrical, suggesting minimal horizontal pleiotropy. Leave-one-out analyses (Fig. 4C) demonstrated that no single SNP disproportionately influenced the overall causal estimates, supporting the robustness of the results. Diagnostic plots for the remaining 32 metabolites were generated but are not shown here, as their trends were similar and sensitivity test results were consistent.

### 3.3. Reverse causal effects of SA on circulating metabolites

To explore the possibility of reverse causality, we conducted MR analyses treating SA as the exposure and the 36 significant metabolites identified in the forward analysis as outcomes. Given the limited number of SNPs reaching genome-wide significance for SA, we adopted a relaxed significance threshold (*P* < 5 × 10⁻⁶) to select IVs. LD clumping was performed using the same parameters as in the forward analysis.

After applying false discovery rate correction, 5 metabolites remained statistically significant: hexadecanedioate (C16-DC; OR = 1.24, 95% CI: 1.04–1.49, *P* = .019), tetradecanedioate (C14-DC; OR = 1.22, 95% CI: 1.02–1.46, *P* = .033), glucuronide of C12H22O4^[1]^ (OR = 0.76, 95% CI: 0.59–0.98, *P* = .036), 2-hydroxyoctanoate (OR = 1.22, 95% CI: 1.01–1.47, *P* = .039), and tryptophan to tyrosine ratio (OR = 1.21, 95% CI: 1.01–1.45, *P* = .044). These findings suggest that SA may exert reverse causal effects on specific metabolic pathways, particularly those related to fatty acid oxidation and aromatic amino acid metabolism.

## 4. Discussion

In this comprehensive bidirectional MR analysis, we evaluated the causal relationships between roughly 1400 circulating plasma metabolites and the risk of SA. The forward MR approach identified 36 metabolites with significant associations to SA risk, showing consistent directional effects across diverse MR methods such as IVW, weighted median, and MR-Egger.

The robustness of these associations was further supported by comprehensive sensitivity analyses, including the MR-Egger intercept test, Cochran’s *Q* test, MR-PRESSO global test, and leave-one-out analysis, all of which indicated no substantial horizontal pleiotropy or heterogeneity. These results imply that the identified associations between metabolites and SA risk are not likely confounded, suggesting true causal relationships.

PAGln, a metabolite derived from gut microbiota processing dietary phenylalanine.^[10]^ Elevated levels of PAGln are associated with adverse cardiovascular outcomes and have been shown to activate adrenergic receptors (α2A, α2B, β2),^[11]^ which enhance platelet reactivity and thrombosis. In the context of pregnancy, excessive adrenergic signaling or a prothrombotic state in uteroplacental circulation could compromise placental perfusion. Our finding that higher PAGln is associated with miscarriage risk suggests a novel gut – vascular axis in pregnancy: gut microbe metabolites like PAGln may contribute to maternal vascular dysfunction or coagulopathy at the fetal – maternal interface, potentially precipitating pregnancy loss. This is consistent with evidence indicating disruptions in metabolic pathways, such as phenylalanine metabolism, in women experiencing recurrent miscarriages.^[12]^ Similarly, the glycerol to carnitine ratio has surfaced as a crucial metabolic marker. Glycerol and carnitine are central to energy metabolism: glycerol reflects lipolysis, while carnitine is essential for β-oxidation of fatty acids. An elevated glycerol: carnitine ratio could indicate inefficiencies in fatty acid utilization or mitochondrial energy production. Notably, our results also hint at immunometabolic crosstalk. A recent MR study in an unrelated inflammatory condition found that a higher glycerol: carnitine ratio causally upregulated human leukocyte antigen-DR isotype expression on CD14⁻ CD16⁻ monocytes,^[13]^ indicating enhanced immune cell activation. Extrapolating to pregnancy, a perturbation in maternal energy metabolism could modulate innate immune function at the maternal – fetal interface. Abnormal activation of monocytes and other antigen-presenting cells, as indicated by elevated human leukocyte antigen-DR isotype expression, has been implicated in pregnancy complications.^[14]^ Thus, the glycerol: carnitine ratio might not only reflect metabolic health but also influence inflammatory tone in early pregnancy. An unfavorable glycerol: carnitine ratio, potentially resulting from carnitine deficiency or excessive lipolysis, may predispose to a pro-inflammatory environment and impaired tissue remodeling at the decidua, increasing miscarriage risk. This interpretation underscores that SA involves a convergence of metabolic and immune dysregulation, as supported by broad evidence that maternal metabolic disturbances often coincide with heightened oxidative stress and “immune response storms” in miscarrying patients^[15]^

1-Methyl-5-imidazoleacetate, a downstream product of histamine metabolism mediated by histamine *N*-methyltransferase and monoamine oxidase, Its association with SA risk underscores the role of histamine and mast cell activity during pregnancy. Histamine is a well-known mediator of allergic and inflammatory responses, and in pregnancy it must be tightly regulated. Excessive histamine can induce uterine contractions and has historically been shown to trigger pregnancy loss in animal models.^[16]^ The placenta normally produces significant amounts of diamine oxidase (DAO), an enzyme that metabolizes histamine, forming a metabolic barrier that protects the fetus.^[16]^ Decreased DAO activity or enhanced histamine release may disrupt this balance.^[16]^ Indeed, reduced DAO activity has been noted in conditions like threatened miscarriage,^[16]^ indicating that an excess of histamine may be harmful. Our results support this notion: elevated levels of a histamine metabolite could signify chronic increased exposure to or metabolism of histamine, suggesting possible histamine intolerance or mast cell activation syndrome in some women experiencing pregnancy loss. The implication is that even subclinical immune activation – via mast cells, basophils, or other histamine-producing cells – might interfere with implantation and placentation. The histamine H4 receptor, for example, modulates immune cell recruitment and cytokine release,^[16]^ and its overactivation could disturb the delicate immune privilege of pregnancy. Thus, the link between 1-methyl-5-imidazoleacetate and miscarriage risk highlights histamine as an immunomodulatory factor in reproductive success. This mechanism, while needing confirmation, aligns with clinical observations that antihistamine therapy or dietary histamine restriction can benefit women with unexplained recurrent pregnancy loss in some cases.

The caffeine to theophylline ratio represents a metabolic marker of caffeine metabolism. Caffeine consumption has long been scrutinized as a miscarriage risk factor, with numerous studies demonstrating a dose-dependent association between higher caffeine intake and pregnancy loss.^[17]^ Our MR results refine this understanding by suggesting genetic variations in caffeine metabolism. A higher caffeine: theophylline ratio suggests slower conversion of caffeine to its metabolite theophylline, presumably reflecting lower CYP1A2 enzyme activity. Interestingly, epidemiologic research shows that fast caffeine metabolizers (higher CYP1A2 activity) are at greater risk of miscarriage when consuming caffeine, compared to slow metabolizers.^[18]^ Specifically, women with high CYP1A2 activity face more than double the odds of miscarriage with moderate caffeine consumption compared to those with low activity (slower metabolism), who do not experience a significant increase in risk.^[18]^ These data suggest that caffeine’s deleterious effects may hinge on its metabolic byproducts or the rate of its clearance. One hypothesis is that rapid metabolism yields higher peaks of certain intermediates (e.g., paraxanthine or theophylline) that could negatively affect the embryo or uteroplacental circulation. Alternatively, genetic proxies for caffeine metabolism might also capture differences in caffeine intake behavior.^[14]^ In our study, a genetic predisposition to a high caffeine: theophylline ratio (potentially denoting slower clearance) was associated with miscarriage, aligning with the notion that caffeine itself is causally harmful. This reinforces public health recommendations to limit caffeine during early pregnancy. Indeed, meta-analyses consistently find that each –150 mg/d caffeine increment (about 1–2 cups of coffee) raises miscarriage risk by around 7% to 19%.^[17,19]^ Our MR adds genetic evidence supporting caffeine’s role, suggesting that women who are genetically or metabolically prone to accumulate caffeine should exercise particular caution.

A strength of our study is the bidirectional MR design, which tested causality in both directions: metabolite effects on miscarriage and miscarriage’s effect on metabolite levels. The forward direction uncovered the 4 metabolites discussed above as putative causal factors. In the reverse direction, we found little evidence that a genetic predisposition to SA leads to altered metabolic profiles. In other words, for these top associations, SA per se did not appear to causally perturb these identified metabolites. This diminishes the concern that our findings are due to reverse causation (e.g., an unrecognized early pregnancy event influencing maternal metabolites). It is more likely that these metabolites (or the pathways they represent) precede and contribute to the pathological cascade resulting in pregnancy loss. We acknowledge, however, that reverse MR analyses were limited by the available genetic instruments for SA. Miscarriage is a complex phenotype with heterogeneous causes, and robust genetic predictors of miscarriage risk are still being identified.^[19]^ The lack of genome-wide significant variants for miscarriage (aside from those related to e.g., progesterone signaling or immune genes) means our reverse MR had limited power. Thus, a true bidirectional relationship cannot be entirely ruled out. For instance, it is conceivable that an unmeasured aspect of miscarriage (such as tissue destruction or stress response) could secondarily alter certain metabolite levels, but our genetic results suggest any such effects are not strong or consistent for the metabolites we identified. By using MR and genome-based instruments, we aimed to strengthen causal inference beyond what observational metabolomics studies can achieve. The use of ICD-based physician-diagnosed SA cases improves classification accuracy; however, the lack of gestational age and individual-level data remains a limitation. This method addresses confounding factors and increases our confidence that these metabolites are not merely epiphenomena, but potentially play an active role in the pathogenesis of miscarriage.^[14]^

## 5. Conclusions

In conclusion, our study provides compelling evidence that metabolic disturbances significantly contribute to the risk of SA. By pinpointing specific metabolites involved in energy metabolism, lipid pathways, and 1-carbon metabolism, we illuminate new aspects of the metabolic underpinnings of pregnancy loss. These findings not only broaden our understanding of how systemic metabolic health impacts reproductive outcomes but also highlight potential metabolic targets for future intervention strategies aimed at preventing miscarriage.

## Author contributions

**Conceptualization:** Xuan Zhou, Yu-Xuan Fang, Da-Wei Zhang, Li-Ya Ma.

**Data curation:** Xuan Zhou, Yu-Xuan Fang, Li-Ya Ma.

**Formal analysis:** Man-Man Yao.

**Investigation:** Li-Ya Ma, Man-Man Yao.

**Methodology:** Xuan Zhou, Man-Man Yao.

**Project administration:** Yu-Xuan Fang, Li-Ya Ma.

**Resources:** Yu-Xuan Fang, Li-Ya Ma, Man-Man Yao.

**Software:** Man-Man Yao.

**Supervision:** Da-Wei Zhang.

**Validation:** Da-Wei Zhang.

**Writing – original draft:** Xuan Zhou, Da-Wei Zhang.

**Writing – review & editing:** Xuan Zhou, Da-Wei Zhang.

## Supplementary Material


